# Tension Pyopneumothorax in an Immunocompetent Adolescent: A Case Report

**DOI:** 10.5811/cpcem.6590

**Published:** 2024-07-18

**Authors:** Elizabeth May-Smith, Marc Olshan, Mark Supino

**Affiliations:** Jackson Memorial Hospital, Department of Emergency Medicine, Miami, Florida

**Keywords:** *tension pyopneumothorax*, *thoracostomy*, *empyema*, *case report*, *pediatrics*

## Abstract

**Introduction:**

Tension pyopneumothorax is a rare, life-threatening condition that occurs as a complication of intrathoracic infection or bronchopleural fistula. In the few cases reported in the literature, the patients typically have multiple comorbidities, underlying lung disease, and/or an immunocompromised state.

**Case Report:**

This case describes tension pyopneumothorax in a previously healthy adolescent male with no existing risk factors for this disease. After emergent stabilization and admission, surgical exploration of the chest revealed no fistulas or pleural defects. Extensive workup did not show any underlying risk factors for development of this condition.

**Conclusion:**

This case of pyopneumothorax with progression to tension physiology is exceedingly rare. Uniquely, the patient had no underlying medical or anatomical predisposition to developing this condition. The case also emphasizes pediatric patients’ capacity to compensate in the setting of critical illness.

CPC-EM Capsule What do we already know about this clinical entity?

*Tension pyopneumothorax is a rare clinical presentation in the emergency department that requires swift recognition and intervention.*

What makes this presentation of disease reportable?

*This is the first described case in an immunocompetent pediatric patient due to the organism* Streptococcus Constellatus.

What is the major learning point?

*Clinicians with suspicion for this presentation should consider large-bore tube thoracostomy and transfer to a facility with pediatric surgical capability.*

How might this improve emergency medicine practice?

*Timely intervention is key to preventing morbidity and mortality. Point-of-care ultrasound and prompt thoracostomy will aid in diagnosis and management.*

## INTRODUCTION

Tension pneumothorax is a standard emergency department (ED) diagnosis, typically seen in the setting of trauma, and requires immediate clinical recognition and intervention. Tension physiology can less commonly occur with other pathology inside the chest, such as infection. Tension pyopneumothorax is a rare, life-threatening condition that can develop in the setting of intrathoracic infection. There are few cases described in the literature. The diagnosis tends to occur in patients with multiple comorbidities and underlying lung pathology.^1^ ^,^ ^2^ We describe a case of tension pyopneumothorax in a previously healthy adolescent with slow development over several weeks. Management includes prompt thoracostomy, respiratory support, antibiotics and, often, surgical management for adequate source control.^3^ ^,^ ^4^

## CASE REPORT

The patient was a 12-year-old Hispanic male who presented to the ED accompanied by his father for shortness of breath. On arrival, the patient was diaphoretic, profoundly tachypneic, and appeared frightened in triage. The father reported that the patient had complained of severe chest pain and difficulty breathing for several days. The patient was promptly moved to the resuscitation room, where initial vital signs were as follows: temperature 37.1° Celsius, heart rate 150 beats per minute, respiratory rate 60 breaths per minute, oxygen saturation 91% on room air, and blood pressure 120/90 millimeters of mercury. He weighed 69 kilograms with a body mass index of 28.

Upon auscultation, absent breath sounds were noted over the left chest. There was initial concern for spontaneous pneumothorax. As the patient was being connected to the monitor and a stat portable chest radiograph (CXR) was ordered, a point-of-care ultrasound demonstrated absent lung sliding over the left chest. No fluid was noted at that time on ultrasound. On further physical exam, the patient was alert with appropriate mentation but appeared to be in significant distress. In the wheelchair before transferring to the stretcher he was in a tripod position and profoundly tachypneic. He was tachycardic with intact peripheral pulse, capillary refill of 2–3 seconds, and no jugular venous distension was noted. The patient’s father elaborated that the patient had complained of shortness of breath and malaise for more than one week leading up to his presentation but denied any cough or fevers. He reported that the patient had no past medical history, no known allergies, and no prior surgeries. The patient was up to date on all vaccinations since immigrating to the United States five years prior from Central America.

**Image 1.**
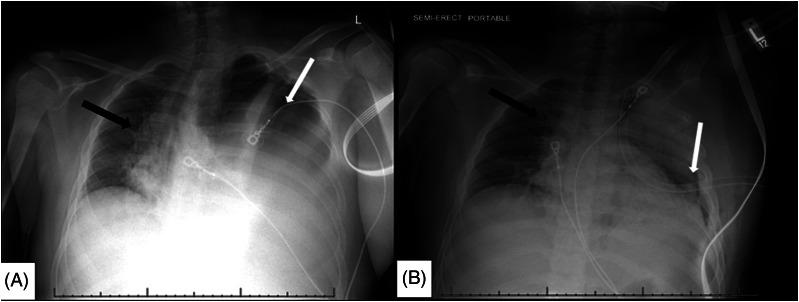
Emergency department chest radiograph (CXR): A) first semi-upright, one view radiograph of the chest demonstrating left-sided hydropneumothorax (white arrow) with shifting of the mediastinal structures toward the right (arrow); B) one view CXR post-procedure demonstrating interval chest tube placement on the left side with expansion of left lung (arrow), improved hydropneumothorax, and resolved tension physiology (black arrow).

A rapid portable CXR demonstrated a left-sided apical pneumothorax with effusion at the base and evidence of tension physiology (Image 1A). Needle decompression was deferred as the patient was normotensive without signs of imminent circulatory collapse, and the team quickly set up for tube thoracostomy. The team was worried that intubation would exacerbate underlying tension physiology and circulatory status; so the patient was maintained on non-rebreather oxygenation and sedated with ketamine for the procedure. A pigtail catheter placement was attempted with lateral approach; however, a syringe of bloody, purulent malodorous fluid was aspirated; thus, the team switched to a traditional large-bore thoracostomy with a 28 French tube. Upon accessing the chest, a large volume of foul-smelling purulent fluid was extruded. A post-procedure radiograph showed the chest tube in proper position and improved position of mediastinal structures, with some persistent left pneumothorax and effusion (Image 1B). The patient’s heart rate improved to 130 beats per minute, respiratory rate decreased to 30 breaths per minute, oxygen saturation of 100% on 15 liters oxygen non-rebreather, and his blood pressure remained the same.

**Image 2.**
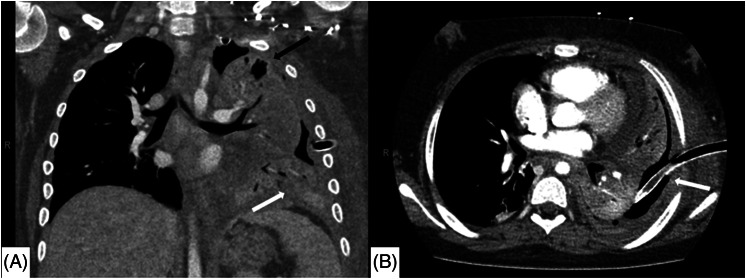
Computed tomography imaging of the chest during early hospital course: A) coronal view and B) axial view demonstrating residual moderate left hydropneumothorax with diffuse opacification of left lung representing extensive pneumonia (white arrow), and peripheral air lucencies on the left suggestive of loculations (black arrow). Chest tube noted to be in good position with fluid and debris obstructing the lumen of tube (white arrow).

Blood work was significant for a white blood cell count of 26,000 per microliter (μL) (normal range 4,500–13,000/μL), lactate of 3.1 millimoles per liter (mmol/L) (0.7–2.1 mmol/L), albumin of 2.8 grams per deciliter (g/dL) (3.9–5.0 g/dL), mild transaminitis, normal renal function, and normal pH on venous blood gas. On further chart review, we found that the patient had been seen in the ED two weeks prior for back pain and had a normal CXR at that time. Broad spectrum antibiotics were administered in the ED, the patient was placed on high-flow nasal cannula, and he was admitted to the pediatric intensive care unit (PICU). By the time he transferred out of the ED, greater than 1.5 liters of purulent output from the chest tube had emptied into the closed chest drainage system. Once the patient was stabilized in the PICU, computed tomography of the chest was obtained the following day that showed a moderate left hydropneumothorax, diffuse opacification of the left lung in the setting of extensive pneumonia, and peripheral air lucencies on the left, likely representing loculations (Image 2).

The patient remained in the PICU for three weeks. He continued parenteral antibiotics and required multiple doses of intrapleural alteplase to clear debris from the chest tube. On hospital day five, he underwent a video-assisted thorascopic (VATS) procedure for washout of the chest and replacement of the original chest tube. Operative report from the VATS procedure described fibrinous debris on the surface of the lung upon entry into the chest cavity, as well as dense adhesions between the lung and pleural surface of the chest wall. Gross examination and recruitment maneuvers did not reveal any other focal abnormalities of the lung itself, and no bronchopleural fistulas were observed.

**Image 3.**
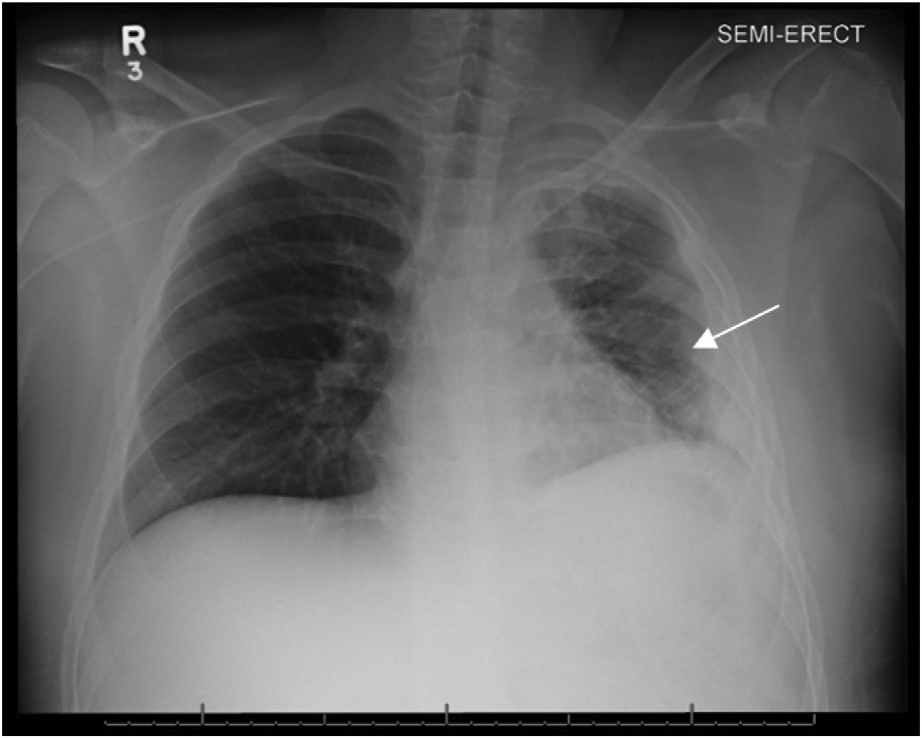
Chest radiograph one view that shows re-expansion of the lung with improved opacification and some residual diffuse interstitial prominence of the left lung (arrow). Interval removal of chest tube.

Culture of the chest tube output grew *Streptococcus constellatus* for which antibiotics were narrowed to ampicillin-sulbactam. The patient required intermittent bilevel positive airway pressure for the first two weeks of admission but was eventually weaned from all supplemental oxygen. Further workup for tuberculosis, underlying immunodeficiency, and malignancy were negative. Ultimately the final CXR demonstrated improved aeration of the left lung (Image 3). The patient was discharged home after a total of one month in the hospital. He attended one follow-up appointment one month after hospital discharge and reported no residual respiratory symptoms at that time. Unfortunately, he was lost to follow-up after that appointment.

## DISCUSSION

Tension pyopneumothorax is a rare, life-threatening condition in which air and pus compress the lung and other intrathoracic structures, and it occurs as a complication of pneumonia, trauma, aspiration, lung abscess, or preexisting bronchopulmonary or pleural fistula.^3^ In the absence of a pleural defect, it is also theorized that a pyopneumothorax can arise from an abscess, empyema, or pyogenic infection with a gas-forming organism inside the chest.^5^ The tension physiology in this case was characterized by severe respiratory distress and signs of shock including tachycardia and mild hypoxia with visible shifting of mediastinal structures on radiograph. Although the patient was normotensive on initial assessment, he became hypertensive throughout hospitalization and required antihypertensive medications upon discharge. This suggests that his presenting blood pressure was likely below his baseline and a sign of early circulatory compromise.

There are few case reports describing this condition, and only a handful of cases are described in the pediatric population. Most documented cases of tension pyopneumothorax have a rapidly progressive clinical course and occurred in patients with underlying disease or immunocompromise, such as malignancy, Parkinson disease, cerebral palsy, substance use, recurrent pneumonias, and/or aspiration events. There is one similar case that describes development of symptoms and progression to tension physiology over several weeks in a pediatric patient.^3^ Our patient’s case is quite unique in that the patient had a gradual development of a pyogenic pneumonia that led to tension physiology without any identifiable underlying disease or immunocompromise.

Anaerobic bacteria are implicated in 30% of pyothorax, although a combined flora can be seen in cases of pyothorax with aspiration etiology.^6^ ^,^ ^7^ *S constellatus* was the culprit organism in our patient’s pyopneumothorax*. S constellatus* is part of the *S anginosu*s group; these anaerobes are native to different systems in the body including the upper respiratory tract, oral cavity, gastrointestinal tract, and reproductive tract.^2^ Since these Gram-positive bacteria are native to the respiratory tract, it can be difficult to distinguish them from contaminant or causative organism on culture; rarely, these bacteria can lead to aggressive pyogenic infections.^8^ *S constellatus* has been associated with abscess formation and empyema. Due to its anaerobic metabolism, *S constellatus* produces gas, which could lead to development of tension in pyogenic intrathoracic infections.^5^

Patients with severe infections typically have risk factors such as extensive smoking history, malignancy, chronic lung disease, periodontal disease, and infectious diseases such as HIV and hepatitis.^2^ One case report describes a 57-year-old male patient with history of hepatitis C, tobacco use, and chronic obstructive pulmonary disease who was found to have an aggressive *S constellatus* cavitary lesion as a sequela of a recent subsegmental pulmonary emboli.^9^ Despite early initiation of broad-spectrum antibiotic coverage, the lesion rapidly expanded, necessitating extensive surgical management including partial pleurectomy and localized resection in addition to antibiotics. Our case is the first to describe *S constellatus* as the causative organism in pyopneumothorax with tension physiology.

The mainstay of treatment for tension pyopneumothorax is pleural drainage and parenteral antibiotics, although for some patients with underlying fistula or other structural abnormalities of the lung, resection or lobectomy may be required.^4^ ^,^ ^10^ Needle decompression is indicated in tension physiology due to impending circulatory collapse; however, this step was not performed in our patient due to normotension and rapid capability to perform tube thoracostomy. Initial pleural drainage in these cases can be done with small- or large-bore tube thoracostomy. Small-bore or pigtail catheters are generally better suited for pneumothoraces and transudative effusions due to their small diameter and potential for occlusion.^11^ In this case, small-bore thoracostomy was attempted but aborted due to aspiration of blood and purulence into the syringe. Even when the patient had successful initial drainage with a large-bore thoracostomy, he eventually required multiple days of intrapleural alteplase due to tube occlusion and definitive VATS procedure to achieve source control. Similar cases presenting to smaller community hospitals would require transfer to a larger center where pediatric thoracic surgery consult and management is available.

## CONCLUSION

Our patient presented with a progressive *S constellatus* pneumonia and empyema that led to formation of a tension pyopneumothorax, a rare, life-threatening condition. This case is unique in that the patient did not have any risk factors for development of the tension pyothorax or severe pyogenic infection from *S constellatus,* and he exhibited a subacute course leading to ED presentation. Additionally, our case highlights the resilience of pediatric patients and their ability to compensate during active disease processes. Our patient presented critically ill from a severe pneumonia that had been developing for at least one week. Despite the tension physiology he developed from the underlying pneumonia, his mental status and blood pressure were intact. The patient did well after initial management with tube thoracostomy and further parenteral antibiotics, supplemental oxygen, and a VATS procedure. The patient might have benefited from earlier surgery, which would have resulted in more rapid source control of such an aggressive infection, but he was ultimately liberated from all respiratory support and completed several weeks of parenteral antibiotics to make a full recovery.

## References

[1] Kanai O Fujita K Okamura M et al . Afebrile tension pyopneumothorax due to anaerobic bacteria: fistula or gas production? Respir Med Case Rep. 2021;32:101372. 33732612 10.1016/j.rmcr.2021.101372 PMC7941028

[2] Noguchi S Yatera K Kawanami T et al . The clinical features of respiratory infections caused by the *Streptococcus anginosus* group. BMC Pulm Med. 2015;15:133. 26502716 10.1186/s12890-015-0128-6 PMC4624190

[3] Whiteman PJ Wilson MT Barcay D et al . Tension pyopneumothorax in a child: a case report. J Emerg Med. 2003;24(4):429–31. 12745046 10.1016/s0736-4679(03)00040-4

[4] Mitsui S Tauchi S . Tension pyopneumothorax. BMJ Case Rep. 2021;14(3):e242197. 10.1136/bcr-2021-242197 PMC7996364 33766977

[5] Hsieh C-F Lin H-J Foo N-P et al . Tension pyopneumothorax. Resuscitation. 2007;73(1):6–7. 17254692 10.1016/j.resuscitation.2006.08.024

[6] Tsang KY Leung WS Chan VL et al . Complicated parapneumonic effusion and empyema thoracis: microbiology and predictors of adverse outcomes. Hong Kong Med J. 2007;13(3):178–86. 17548905

[7] Jerng J-S Hsueh P-R Teng L-J et al . Empyema thoracis and lung abscess caused by viridans streptococci. Am J Respir Crit Care Med. 1997;156(5):1508–14. 9372668 10.1164/ajrccm.156.5.97-03006

[8] Porta G Rodriguez-Carballeira M Gomez L et al . Thoracic infection caused by *Streptococcus milleri* . Eur Respir J. 1998;12(2):357–62. 9727785 10.1183/09031936.98.12020357

[9] Vulisha AK Sam R Nur H et al . Aggressive presentation of *Streptococcus constellatus* . Cureus. 2021;13(4):e14534. 34017651 10.7759/cureus.14534 PMC8128152

[10] Reid JM Barclay RS Stevenson JG et al . The management of spontaneous pyopneumothorax and empyema in young children. Dis Chest. 1966;49(2):175–8. 5907973 10.1378/chest.49.2.175

[11] Anderson D Chen S Godoy L et al . Comprehensive review of chest tube management. JAMA Surg. 2022;157(3):269–74. 35080596 10.1001/jamasurg.2021.7050

